# Barriers to Low-Dose CT Lung Cancer Screening among Middle-Aged Chinese

**DOI:** 10.3390/ijerph17197107

**Published:** 2020-09-28

**Authors:** Qike Jia, Hongliang Chen, Xuewei Chen, Qichuan Tang

**Affiliations:** 1 School of Management, Zhejiang University of Technology, Hangzhou 310023, China; qikejia@zjut.edu.cn; 2 College of Media and International Culture, Public Diplomacy and Strategic Communication Research Center, Zhejiang University, Hangzhou 310058, China; 3 School of Community Health Sciences, Counseling and Counseling Psychology, Oklahoma State University, Stillwater, OK 74078, USA; xuewei.chen@okstate.edu; 4 College of Media and International Culture, Zhejiang University, Hangzhou 310058, China; 3170105826@zju.edu.cn

**Keywords:** low-dose CT, lung cancer screening, prevention

## Abstract

Purpose: The current study aims to explore the barriers for middle-aged Chinese to learn about and uptake low-dose computed tomography (LDCT) lung cancer screening. Methods: Data were collected via an online survey in December 2019. Final valid sample included 640 respondents, aged 40–60 years old, from 21 provinces of China. We performed multiple linear regressions to test the potential barriers to LDCT scan. Findings: Cost concerns, distrust in doctors, fears of disease, lack of knowledge, and optimistic bias are negatively associated with the intention to learn about and uptake LDCT scan. Implications: Our study contributes to understanding the negative predictors of middle-aged Chinese to get LDCT lung cancer scans. Future campaign programs should help audiences to build comprehensive understandings about lung cancer and LDCT scan. To better promote LDCT scan in China, the government should fund more trial programs continuously and public efforts should be made to rebuild the patient–doctor trust.

## 1. Introduction

Lung cancer is the leading cause of cancer mortality in China [1]. A survey in 2015 indicated that lung cancer accounts for 22 percent of China’s cancer deaths [2]. From 1975–2015, the confirmed mortality cases increased by 465 percent [3]. Research suggested that the risk of lung cancer began to rise at the age of 30–40 and peaked for those who were 80 years and over [4]. The lung cancer incidence rate in China is about three times higher than the United States [5]. In China, lung cancer incidence has grown in younger people. According to a recent study, lung cancer occurred more frequently in patients who are older than 35 years in China than ever before [6]. The majority of middle-aged patients were diagnosed at the late stage of lung cancer [7]. Smoking, second hand smoking exposure, indoor air pollution, prior lung diseases, and family cancer history are the main attributes of lung cancer in China [8,9]. Moreover, the frequent use of coal burning for cooking [10,11], severe air pollution [12], work history in construction site [13], and unhealthy diet [14] also contribute to the increasing lung cancer mortality. Patients, their families, and the healthcare system all suffered the grieving pain of high treatment costs. A prior study documented that the average cost of lung cancer treatment was $43,336 per patient, which is ten times the annual dispensable income per capita in China [15,16].

In China, the government plays a pivotal role in funding public hospitals and cutting the cost of medical services [17]. In response to the alarming death numbers and the increased economic burdens to patients and family, in 2012, the central government of China initiated a large-scale lung cancer screening trial in both rural and urban areas nationwide [18]. Since then, the government has announced plans to build more lung cancer screening facilities targeting high-risk populations, which originated from 9 provinces and later extended to 18 [19]. Public health campaigns were conducted targeting residents of major metropolitan areas to raise public awareness of lung cancer issues. However, there are still concerns about the under coverage of cancer test services and the quality of cancer treatment outcomes [20]. As most healthcare providers are located in big cities, the cancer mortality rate in rural areas of China is much higher than the urban counterparts [21]. The unbalanced distribution of cancer service supply, unstandardized insurance coverage, and lack of qualified radiologists impede disadvantaged populations in accessing to lung cancer treatments [22,23].

Essential for reducing lung cancer mortality is early screening. Patients of lung cancer often stay asymptomatic for years until the cancer has advanced and disseminated [24]. The five-year survival rate decreases dramatically when the cancer presents in a later stage, excluding the patients from curative surgery [25]. The data of the American Cancer Society indicate that the five-year survival rate for non-small cell lung cancer was 61% if diagnosed in the localized stage (cancer has not spread outside of the lung), compared to 35% during the regional stage (spread to lymph nodes or other nearby structures) and 6% during the distant stage (spread to distant parts of the body) [26]. If lung cancer is found at an earlier stage, it is more likely to be successfully treated.

Fortunately, the introduction of low-dose computed tomography (LDCT) advanced the early diagnosis significantly. This newer technology, using three-dimensional X rays, can identify small abnormalities in human bodies [27]. In the United States, researchers found that regular LDCT lung cancer screening reduced the mortality rate, especially to the high-risk population, such as current and former smokers [28]. However, compared with traditional chest CT scan, the cost of LDCT is much higher [29]. Since 2018, LDCT has been covered by medical insurance for the inpatient treatment in many cities around China [9]. Though partially funded by the government, the cost concerns about the initial scan and the follow-ups remain to be one of the major barriers to scanning. The current study aims to explore the barriers to LDCT among middle-aged Chinese, which could provide valuable implications for lung cancer prevention in China.

Protection motivation theory (PMT) proved to be an effective framework to predict individuals’ cancer screening behaviors. PMT was developed to understand people’s responses to fear appeals [30,31]. PMT describes that people’s coping strategies of a health threat consists of two types of cogitive appraisal: threat appraisal (perceived severity, perceived vulnerability, perceived rewards) and coping appraisal (response effecicacy, self-efficacy, and response costs) [32]. PMT has been used to test people’s intention to engage in cancer prevention behaviors (i.e., prostate cancer screening [33], breast cancer screening [34], and cervical cancer screening [35]). Drawing from the construct of response cost in PMT, we aim to examine the effects of cost concerns, access challenges, and lack of knowledge on the intention to learn about and take up LDCT lung cancer screening. The core logic of PMT is that individuals undergo comprehensive appraisal of health threat before behavioral changes. However, individuals are sometimes irrational in cancer control and prevention, of which the perceptual bias could result in the ignorance of lung cancer screening. To fill the literature gap, we tested the effects of such biased perceptions, including distrust in providers, fear of disease, and optimistic bias.

### 1.1. Barriers to LDCT Lung Cancer Screening

#### 1.1.1. Cost Concerns

Data showed that the cost of an LDCT scan in China is around US $80, which is covered by insurers and sponsored by the government [3]. Still, this poses a financial burden in a developing country. The gap between coverage policy and patients’ cost concerns poses barriers to lung cancer screening. In addition to the fears about extra expenses, a previous study conducted in the United States found that the expected time taken from work and the unfamiliarity with insurance policy lower people’s intention to take an LDCT scan [36]. The concerns about financial burden on follow-up scans and the travel expenses further increase individuals’ economic pressure [37].

In China, middle-aged adults are facing the pressure of child education, parent healthcare, and the rising prices of housing and living expenses. Lack of health insurance coverage and potential coverage denial are likely to increase people’s cost concerns about the LDCT scan. Moreover, the concerns could extend to the following medical treatments. The burdens above could lower the intention of middle-aged Chinese to take up an LDCT scan.

#### 1.1.2. Access Challenges

A prior study in the United Kingdom indicated that travel distance between place of residence and health providers, lack of public transit connection, and travel costs were most common reasons for refusing screening [38]. The travel distance adds another obstacle to LDCT scanning. The lack of LDCT scanners, professional radiologists, and healthcare facilities limit people’s access to lung cancer screening [3]. In China, a country with unbalanced socioeconomic development, the majority of screening facilities are clustered in the major metropolitan areas, which increases the access challenges among those living in rural areas or small cities [1,2]. Even in the major cities, the worsened traffic, long waiting time, and busy work schedule can be predictors of missing scan appointments. According to a recent survey, the national average work time in China is 46.3 h per week [39]. For those working in the information technology industry, it is common to work from 9 a.m. to 9 p.m., six days a week [39]. The exhausted workers may not be willing to take up any cancer scan after a rough week. Additionally, in the hospitals with LDCT scanners and registered radiologists, the programs are operating at full capacity [3], which can lead to prolonged waiting time and consequently decrease access.

#### 1.1.3. Distrust in Providers

Different from Western countries, doctors are not well-respected in China. During the past decades, China’s healthcare system transformed from a government-funded mode to a fee-on-service mode. Patients tend to believe that hospitals always seek ways to facilitate unneeded tests, overprescribe medications, and raise the inpatient service fees [40]. Patients feel that they end up paying for unnecessary medical procedures [41]. In rural areas, hospitals lack advanced equipment and experienced physicians, which explains why rural patients flock to hospitals in big cities [22].

In the mass media of China, doctors and hospitals are often portrayed as greedy and incompetent. In the case of lung cancer prevention, patients who lack trust in doctors and hospitals are likely to oppose a doctor’s recommendation to have LDCT scanning. Such novel technology can be perceived as a new trick to increase expenses. Middle-aged Chinese who grew up in an era (1960s–1980s) in which healthcare facilities were fully funded by government are likely to be critical and skeptical of the current commercial healthcare mode. The distrust in hospitals and doctors, especially the concern about unnecessary expenses, is likely to lower their intention to have the LDCT scan.

#### 1.1.4. Fears of Disease

Unlike perceived severity (a core construct of PMT) focusing on the threat of a disease on one’s health, fear of disease in this study refers to the concerns about the negative influences of disease on one’s normal life. An individual who possesses high fear of disease is likely to avoid any lung cancer test because of the fear that their life will be ruined if confirmed. The negative attitudes about cancer diagnosis and stigma attached to it can cause patients’ avoidance of screening. For instance, a prior study indicated that smokers are fearful about being diagnosed with lung cancer and unwilling to engage in any conversations about it [42]. Moreover, current and former smokers tend to panic about the uncertainty of scan results and the follow-up treatments [36]. In China, a typical collectivist society, middle-aged people took responsibilities to take care of the children and their parents. The fear about losing jobs and sudden medical expenses can lower their intention to face the challenge of lung cancer upfront, which is likely to add another obstacle to LDCT scanning.

#### 1.1.5. Lack of Knowledge

In China, the general public are not aware of the risks of lung cancer. Most people notice the high prevalence of lung cancer but are lacking in knowledge about the symptoms of disease, effective treatments, and prevention methods. While feeling healthy, Chinese people consider visiting hospitals as a taboo [3]. In addition, people from China tend to believe that cancer is uncurable and no treatment means is effective [43]. The uncertainty about incurring treatment expenses and follow-up tests causes more fears about the novel technology of lung cancer scanning [9]. The limited knowledge can result in the refusal to undergo LDCT screening.

#### 1.1.6. Optimistic Bias

Optimistic bias refers to people’s beliefs that their chances of experiencing a negative event is lower than other people [44]. People with high optimistic bias are likely to maintain unhealthy behaviors and perceive preventive test as unnecessary [45,46]. The long work hours and busy schedule, abetted by the optimistic bias, could lower the intention of middle-aged Chinese to take LDCT tests.

In the current study, we hypothesize that cost concerns, access challenges, distrust in doctors and hospitals, fear about disease, lack of subjective knowledge, and optimistic bias pose barriers to middle-aged Chinese to uptake LDCT lung cancer scans. Given that the LDCT scan has been introduced to China for only a few years, most people may not be familiar with the scan. We tested two types of behavioral intention regarding LDCT scan: (1) the intention to learn about the LDCT scan; (2) the intention to take up an LDCT scan regularly. The findings of this study could contribute to providing solutions to related health campaign nationwide improving lung cancer screening.

## 2. Methods

### 2.1. Procedures and Participants

Data were collected using an online survey between 24–30 December 2019. We used SoJump, one of the largest online survey platforms in China, to recruit participants nationally in China. The survey was approved by the Institutional Review Board at Zhejiang University. SoJump sent 676 invitation emails to eligible participants aged between 40 and 60 years in mainland China. Each respondent (*n* = 660, response rate = 97.6%) received a 12 RMB gift card from SoJump after completing the survey. We dropped the duplicate cases (repetitive submission using the same IP address) and those respondents whose ages were not in the assigned range (40–60 years old). The number of qualified respondents was 640, and they came from 22 provinces of China (56.67% of the respondents were male). According to Cochran’s sample size formula [47], if the alpha level is set at 0.05 and the level of acceptable error is at 3%, the sample size of this study (*n* = 640) could provide satisfactory statistical power.

The average age of respondents was around 48. Respondents’ educational attainment is as follows: elementary school (0.6%), middle school (4.8%), high school (11.6%), associate degree (30.8%), bachelor’s degree (46.9%), and graduate degree (5.3%). The monthly household income was as follows: below 5000 RMB (10.5%), 5001–12,000 RMB (38.1%), 12,001–25,000 RMB (30.8%), 25,001–35,000 RMB (10.8%), 35,001–55,000 RMB (6.7%), 55,001–80,000 RMB (1.7%), and above 80,001 RMB (1.4%). Most respondents (98.3%) were from urban areas (four major metropolitan areas including Beijing, Shanghai, Guangzhou, and Shenzhen = 30.9%; provincial capital cities = 28.7%; prefecture-level cities = 23.9%; county-level and township-level cities = 14.7%; villages = 1.4%). A third of respondents claimed to have a smoking history and 13.1% had family lung disease history. Most respondents (95.5%) had health insurance coverage. As people with chronic diseases are likely to take health tests regularly, we tested respondents’ disease burden using a 7-item scale, including respiratory (i.e., asthma), circulatory system (i.e., high blood pressure), digestive system (i.e., gastritis), endocrine (i.e., thyroid disease), metabolic (i.e., diabetes), psychological and bone diseases. Responses were given using a 5-point scale from 0 “no symptoms” to 4 “severe symptoms” (M = 1.64, SD = 0.58). Descriptive statistics of control variables are displayed in Table 1.

### 2.2. Measures

#### 2.2.1. Cost Concerns

Prior studies noted that individuals’ cost concerns were not limited to the lung cancer scanning itself but also include the expected time loss. In the current study, we used two items to measure respondents’ cost concerns about LDCT screening: “I think the LDCT lung cancer screening is too expensive” and “I think LDCT scan takes up too much time, which impacts my life and work”. Responses were given with a 5-point scale from 1 “strongly disagree” to 5 “strongly agree”. We computed the mean values for these two items, of which higher values indicated greater cost concerns (M = 2.12, SD = 0.99; Cronbach’s α = 0.79).

#### 2.2.2. Access Challenge

We used a single item to assess respondents’ challenge in getting access to LDCT lung cancer scan service (“I think the LDCT screening site is too far from my place of residence”). Responses were given from 1 “strongly disagree” to 5 “strongly agree” (M = 2.06, SD = 1.10).

#### 2.2.3. Distrust in Hospitals

Adapted from a prior study [48], we used five statements to examine respondents’ distrust in hospitals. Items included “the accuracy of hospital lung cancer screening is questionable,” “hospitals put making money above patients’ needs,” “hospitals tend to add unnecessary testing items into lung cancer screening,” “equipment used for lung cancer screening is outdated,” and “screening procedures were not well-designed”. The options ranged from 1 “strongly disagree” to 5 “strongly agree”. We calculated mean values of the items for distrust in hospitals (M = 2.84, SD = 0.87; Cronbach’s α = 0.81).

#### 2.2.4. Distrust in Doctors

We used five items to measure respondents’ distrust in doctors. Items included “doctors in my city cannot provide accurate diagnosis,” “…lack professional knowledge,” “…lie to make money,” “…do not listen to patients’ needs,” and “…rely on their experiences too much.” Respondents were asked to self-report their attitude to the items, ranging from 1 “strongly disagree” to 5 “strongly agree”. We computed the average value of the five items to represent distrust in doctors (M = 0.29, SD = 0.92; Cronbach’s α = 0.85).

#### 2.2.5. Fears of Disease

Three items were used to examine respondents’ fear of disease. Items included “once feel uncomfortable, I will choose not to see doctors for fear that the disease will ruin my normal life,” “…the disease is serious,” and “…the disease symptom will last for a long time.” Similarly, the responses were from 1 “strongly disagree” to 5 “strongly agree”. We averaged the responses and higher scores represent greater fears of disease (M = 2.75, SD = 1.04; Cronbach’s α = 0.76).

#### 2.2.6. Lack of Knowledge

We created ten factual statements about the symptoms and treatments of lung cancer (i.e., “blood in sputum is one of the typical early manifestations of lung cancer,” “one of the common symptoms of a cough that lasts for a long time,” and “the preferred treatment for early lung cancer is surgical resection rather than drug treatment”). Responses were given from 10 (do not know at all) to 1 (know well). We averaged the ten items to represent lack of knowledge (M = 6.30, SD = 1.66; Cronbach’s α = 0.87). The higher values represent the higher level of unknowing.

#### 2.2.7. Optimistic Bias

Consistent with prior study [44], we asked respondents to estimate the chance of being diagnosed with lung cancer and other lung diseases in their lifetime. Similarly, they were asked to report the chances of others in their age range who live in the same city. Responses were given from 1 “very unlikely” to 5 “very likely.” We found that perceived odds of developing cancer in the lifetime is higher on others than themselves, providing support for the existence of optimistic bias. Values on others were subtracted by values on self to create the optimistic bias (M = 0.48, SD = 1.12).

#### 2.2.8. Intention to Learn about and Have an LDCT Scan

We assessed two types of behavioral intention: intention to learn more about the LDCT scan and the intention to take up the LDCT scan. Responses were given from 1 “strongly disagree” to 5 “strongly agree.” Higher values indicate greater behavioral intention (intention to learn about the scan: M = 3.55, SD = 1.03; intention to have the scan: M = 3.02, SD = 1.20). See descriptive statistics of independent and dependent variables in Table 2.

### 2.3. Data Analysis

We performed multiple linear regressions to identify the barriers to LDCT lung cancer scanning. Two types of behavioral intention regarding LDCT scan were examined: (1) intention to learn about the LDCT lung cancer scan (See Models 1 and 2 in Table 3); (2) intention to have the LDCT lung cancer scan (See Models 3 and 4 in Table 3). Models 1 and 3 did not include control variables but Models 2 and 4 did. Given that sociodemographic factors (i.e., age, gender, education, and income) can potentially impact the behavioral intention to learn and uptake the scan, we added these variables as control variables. Moreover, the existing research suggests that substantial disparities in healthcare quality exist between rural and urban areas and between coastal provinces and inland provinces across China [17]. Recent data indicated that cancer mortality and 5-year survival rate are much worse in rural areas than urban areas; and similarly, related data were worse in western and southwestern provinces than in their eastern counterparts [9]. Chinese people often trust hospitals in big cities (i.e., Beijing and Shanghai) more for cancer treatment consultation. Given that the size of city in residence could impact the chances to access LDCT scan, we added the city size as a control variable.

Besides sociodemographic factors and city size, we added some other control variables that were found to be predictors of lung cancer screening in previous studies, including smoking history [8], family genetic factors [49], insurance coverage status [50], and health status (disease burden) [51] as controls.

## 3. Results

In the current study, we proposed that cost concerns, access challenge, distrust in hospitals, distrust in doctors, fear of disease, lack of knowledge, and optimistic bias were the likely barriers for middle-aged Chinese to take up LDCT lung cancer screening. We measured the variance inflation factor (VIF) to assess the multicollinearity. The value of VIF in the regression models (See Table 3) were all below 3.0, which is acceptable according to a previous study [52]. Holding constant for control variables, the results indicated that cost concerns (β = −0.9, *p* < 0.001), fear of disease (β = −0.10, *p* < 0.01), lack of knowledge (β = −0.18, *p* < 0.001), and optimistic bias (β = −0.08, *p* < 0.05) negatively predicted the intention to learn about LDCT ng (See Model 2 in Table 3). Next, the results indicated that cost concerns (β = −0.23, *p* < 0.001), distrust in doctors (β = −0.13, *p* < 0.05), lack of knowledge (β = −0.20, *p* < 0.001), and optimistic bias (β = −0.18, *p* < 0.001) were negative predictors of the intention to uptake LDCT scan (See Model 4 in Table 3). The effects of access challenge and distrust in hospitals were not significant. Moreover, we conducted sensitivity analyses with the older sample (aged between 50 and 60). The results of the regression models did not change.

Additionally, we found that age, household income, education, city size, smoking history, insurance coverage and disease burden positively predicted the intention to learn about or have the LDCT scan (See Models 2 and 4 in Table 3). The results indicated that low socioeconomic status, lack of insurance coverage, and living in smaller cities pose barriers to access to LDCT scan.

## 4. Discussion

The current study explored the potential barriers to accessing the LDCT lung cancer scan among middle-aged Chinese. We found that cost concerns lowered individuals’ intention to learn about and take up the LDCT scan. This finding is consistent with the PMT framework [36,37,53,54], suggesting that response cost inhibits individuals to engage in behavioral change. The concerns about expenses in initial scan, follow-up treatment, and potential time taken away from work are likely to impede middle-aged Chinese to undergo the LDCT scan. In the past four decades, China has achieved economic success but the annual income per capita is still far behind Western countries, especially in the underdeveloped areas [55]. For middle-aged Chinese, their priority is to cover the high costs in children’s education and finance aging parents’ healthcare needs [56]. Middle-aged people may also be concerned about career development, rising living expenses, and high housing prices, but little attention is paid to their own lung cancer risks [56]. Moreover, a reform in China has driven the healthcare system from a government-funded model to a commercial service model [57]. Even though the government has implemented trial programs to fund the LDCT scan and expanded the insurance coverage in both urban and rural areas, the public may be unaware of these new programs and still hold the opinion that novel technology in lung cancer scanning could be very expensive. Our study indicated that the strategies to improve LDCT lung cancer scanning should not be limited to lowering the costs of scan but to educate the public the presence of state-funded programs via multiple media channels.

Second, people’s biased perceptions (i.e., distrust in doctors and hospitals, optimistic bias, and fear of disease) were found to lower their intention to uptake the LDCT scan, indicating that people often do not follow the logic of PMT in the face of lung cancer threat. In our study, about half of respondents claimed that they do not trust physicians or hospitals. In contrast, taking the UK as an example, a recent survey indicated that nine out of ten patients have confidence in their doctors, nurses, and pharmacists [58]. Our study endorsed the fact that compared to Western countries, Chinese people do not trust their healthcare providers. Distrust in healthcare providers has led to increased tensions between patients and doctors [59]. A survey indicated that in 2010 alone, there were over 17,000 cases of violence against doctors in China [60]. Patients tend to think that doctors put making money before patients’ health [61]. The negative consequence of distrust in doctors is that patients’ refusal to follow physicians’ clinical treatment comes at the cost of health deterioration. When suggested to uptake an LDCT scan, Chinese patients are likely to question the purpose of the doctor’s recommendation and refuse to take the scan. To promote LDCT scanning in China, rebuilding patient–doctor trust is key. Additionally, the government sectors, non-government organizations, and mass media should work together to inform the public about LDCT scanning.

Third, we found that the optimistic bias and lack of knowledge were negatively associated with the intention to take up the LDCT scan. The results indicated that respondents were unfamiliar with lung cancer symptoms, screening, and treatment, but in the meantime, they tended to be optimistic about their lung cancer risk. The similar low awareness of lung cancer threat was also documented in Western countries [62,63,64]. Prior studies also found that adults and adolescents with high optimistic bias were likely to believe that their smoking history would not lead to any serious health problems [65,66]. The underestimated risk perception of lung cancer could result in the unawareness of preventive measures that can effectively reduce the chances of disease development, such as regular LDCT scanning. The misperceptions about lung cancer that have resulted in the continuation of risky behaviors and inadequate preventive measures might account for the rising prevalence of lung cancer in China.

Additionally, we found that fear of disease also lowered one’s intention to learn about LDCT scan. Unlike the pessimistic perception, those who fear about lung cancer are likely to be excessively panicked to learn about lung cancer and preventive strategies. In Chinese culture, it is a taboo to talk about death and diseases between family members [67]. The economic burdens on middle-aged people intensifies the anxiety about the negative consequences after being sick. Such cultural identity in China raises people’s concerns about being diagnosed with lung cancer. The findings of our study suggested that to promote the use of LDCT scanning, health campaigns should aim to inform the public that the early detection of lung cancer (i.e., LDCT scans) is essential to improve survival rate and quality of life. To reverse taboo and misperceptions about cancer in Chinese culture, LDCT scanning should be strategically portrayed as a guard, not an alarm, to their health.

### Limitations

The survey data used are cross-sectional, which cannot identify causal inferences. Later work should consider collecting data at multiple time points to identify the trend of LDCT scan adoption in China. Second, China is a vast country with unbalanced social and economic development. Significant disparities exist between urban and rural areas as well as the developed and underdeveloped areas. The insignificant effect of access challenge in this study can be attributed to the fact that most respondents were from urban areas. Future study focusing on comparison between different populations could provide more valuable implications about future lung cancer screening promotion. The survey data used in the current study cannot fully represent Chinese people living in the rural areas and small towns, which might lead to biased conclusions. Third, our study did not examine the effects of media use on behavioral intention to uptake an LDCT scan. As media played an important role in health communication, future studies should assess how different media outlets influence the audience’s perceptions about lung cancer and preventive measures.

## 5. Conclusions

Our study contributes to understanding the negative predictors of middle-aged Chinese people’s access to the LDCT lung cancer scan. We found that cost concerns, distrust in doctors, lack of knowledge, optimistic bias, and fears of disease pose barriers to learning about and taking up the LDCT scan. Future campaign programs should aim to increase public awareness of the preventive measures for lung cancer and reverse the cultural taboo about open conversations regarding diseases. Public efforts should be made to rebuild the patient–doctor trust in China and fund more LDCT scan programs nationwide.

## Figures and Tables

**Table 1:** Descriptive Statistics (N = 640).

Variables	Mean/Percentage	SD	Min	Max
**Control Variables**				
Gender (male) ^a^	56.67%			
Age	48.02	5.07	40	60
Household income	2.76	1.23	1	7
1 = Below 5000 RMB	10.5%			
2 = 5001–12,000 RMB	38.1%			
3 = 12,001–25,000 RMB	30.8%			
4 = 25,001–35,000 RMB	10.8%			
5 = 35,001–55,000 RMB	6.7%			
6 = 55,001–80,000 RMB	1.7%			
7 = Above 80,001 RMB	1.4%			
Educational Attainment	5.34	0.96	2	7
1 = No education experience	0%			
2 = Elementary school	0.6%			
3 = Middle school	4.8%			
4 = High school	11.6%			
5 = Associate degree	30.8%			
6 = Bachelor degree	46.9%			
7 = Graduate degree	5.3%			
City Size	3.73	1.10	1	5
1 = Villages	1.7%			
2 = County-level/Township-level cities	14.7%			
3 = Prefecture-level cities	23.9%			
4 = Provincial capital cities	28.7%			
5 = Beijing/Shanghai/Guangzhou/Shenzhen	30.9%			
Current or former smokers ^a^	33.59%			
Family medical history ^a^	13.12%			
Health insurance coverage (yes) ^a^	95.47%			
Disease burden	1.64	0.58	1	4.29

^a^ Represents the frequency of a dichotomous variable.

**Table 2:** Descriptive Statistics of Independent and Dependent Variables (*N* = 640).

Variables	Mean	SD	Min	Max
**Dependent variables**				
Intention to have LDCT	3.02	1.20	1	5
Intention to learn about LDCT	3.55	1.03	1	5
**Independent variables**				
Cost concerns	2.12	0.99	1	5
Access challenges	2.06	1.10	1	5
Distrust in hospitals	2.84	0.87	1	4.80
Distrust in doctors	2.95	0.92	1	5
Fear of disease	2.75	1.04	1	5
Lack of knowledge	6.30	1.66	1.70	10
Optimistic bias	0.48	1.12	−3	4

**Table 3:** Standardized Ordinary Least Squares Regression predicting intention to learn about and uptake Low-Dose Computed Tomography (LDCT) Lung Cancer Screening (*N* = 640).

Variables	Model1 ^a^	VIF ^c^	Model2 ^a^	VIF	Model3 ^b^	VIF	Model4 ^b^	VIF
**Main Effects**								
Cost concerns	−0.19 ***	1.47	−0.19 ***	1.48	−0.22 ***	1.47	−0.23 ***	1.48
Access challenges	−0.06	1.44	−0.06	1.49	0.00	1.44	−0.01	1.49
Distrust in hospitals	−0.08	2.50	−0.05	2.53	0.02	2.50	0.06	2.53
Distrust in doctors	0.01	2.42	−0.01	2.45	−0.11 †	2.42	−0.13 *	2.45
Fears of disease	−0.12 **	1.16	−0.10 **	1.21	−0.09 *	1.16	−0.07 ^†^	1.21
Lack of knowledge	−0.22 ***	1.01	−0.18 ***	1.06	−0.22 ***	1.01	−0.21 ***	1.06
Optimistic bias	−0.12 **	1.01	−0.08 *	1.15	−0.20 *	1.01	−0.18 ***	1.15
**Controls**								
Gender (male)			−0.06	1.39			−0.03	1.39
Age			0.08 *	1.07			0.06 ^†^	1.07
Household income			0.10 *	1.35			0.07 ^†^	1.35
Educational attainment			0.10 **	1.20			0.07 ^†^	1.20
City size			0.00	1.24			0.07 ^†^	1.24
Smoking history			0.09 *	1.48			0.02	1.48
Family medical history			0.06	1.07			0.02	1.07
Insurance coverage			0.06 ^†^	1.04			0.07 *	1.04
Disease burden			0.08 *	1.15			0.11 **	1.15
Adjusted R Squared	0.09 ***		0.21 ***		0.07 ***		0.21 ***	

Note. † *p* < 0.10, * *p* < 0.05, ** *p* < 0.01, *** *p* < 0.001. ^a^ DV = intention to learn about LDCT scan; ^b^ DV = intention to uptake LDCT scan; ^c^ VIF stands for Variance Inflation Factor.
